# Neuroimaging of Brain Tumor Surgery and Epilepsy

**DOI:** 10.3390/brainsci13121701

**Published:** 2023-12-10

**Authors:** Takehiro Uda

**Affiliations:** Department of Neurosurgery, Osaka Metropolitan University Graduate School of Medicine, 1-4-3, Asahi-machi, Abeno-ku, Osaka City 545-8585, Osaka, Japan; udat@omu.ac.jp

To make the best clinical judgements, surgeons need to integrate information acquired via multimodal imaging. For brain tumor surgery, the tumor’s location, size and origin (intra-axial or extra-axial) should be examined with preoperative computed tomography and structural magnetic resonance imaging (MRI). The precise estimation of a tumor’s pathology is also important. For surgical treatment, surrounding structures such as vessels, nerves, eloquent cortices and related white matter tracts should be evaluated to avoid unwanted damage due to surgery. Thereafter, the best surgical approaches should be selected. Recently, endoscopic surgery has been introduced and is becoming popular, especially in the fields of pituitary surgery [1,2] and skull base surgery [3,4]. For epilepsy surgery, in addition to these structural imaging techniques, neurophysiological information acquired via electroencephalography and magnetoencephalography and functional information acquired via positron emission tomography (PET) are necessary for decision making. The surgical goal in epilepsy surgery is to control the patient’s seizures and enable them to continue their usual daily activities. Then, in addition to surgery with craniotomy, neuromodulation surgeries such as vagus nerve stimulation, deep brain stimulation, and responsive neurostimulation are considered on a patient-by-patient basis [5].

The main aims of this Special Issue, entitled “Neuroimaging of Brain Tumor Surgery and Epilepsy”, were to collect clinical articles about preoperative and postoperative neuroimaging and to share the rare but educational case reports based on clinical experiences. As a result, two research articles and four case reports were published in this Special Issue.

First, Takayama Y et al. published a research article about surgical strategies and seizure and neurocognitive outcomes for low-grade epilepsy-associated tumors (LEATs) on the temporal lobe. Surgical strategies for LEATs and the consensus regarding hippocampal resection are still widely debated in epilepsy surgery [6,7,8,9,10]. They concluded that additional hippocampal resection might result in postoperative language dysfunction. This article offered surgeons a new insight into clinical judgement when removing LEATs on the temporal lobe [11].

Second, Uda H et al. published a research paper about the visualization of resected areas for skull base meningioma, comparing conventional transcranial microsurgery [12,13,14] and endonasal endoscopic surgery [15,16,17]. For the visualization, they used a novel computational method called “Voxel-Based-Lesion Mapping”. They clearly demonstrated a border between transcranial surgery and endoscopic endonasal surgery. The border closely matched a circle connecting the neural foramens (optic canal, foramen rotundum, foramen ovale, internal auditory canal, jugular foramen and hypoglossal canal). Their results might be helpful for surgeons when selecting better surgical approaches [18].

Nakae S et al. published a case report regarding the usefulness of depth electrodes used as fence posts. The role of fence posts is to make a margin of removal in brain tumor and epilepsy surgery. Conventionally, various kinds of rubber or plastic tubes are utilized as fence posts, which are usually inserted under the guidance of neuronavigation [19,20,21,22]. Not only do they have a conventional role as an anatomical landmark, but they also serve as electrophysiological information to fence posts. Particularly when operating on low-grade gliomas, surgeons should consider the control of seizures in addition to the tumor removal when making a surgical decision. Their method might offer important intraoperative information for better seizure outcomes [23].

Kawashima T et al. published a case report about rare postoperative hemorrhagic complications after stereotactic electroencephalography (SEEG). Hemorrhagic complications are the main concerns after SEEG [24,25,26,27,28]. Previously, among various types of hemorrhagic complications, intraparenchymal hemorrhage had generally been thought to be the result of direct vessel injury caused by a puncture needle. However, the authors’ postoperative imaging suggested that the location of the damaged artery was far from the puncture needle. They speculated that the cause of the hemorrhage was the excessive stretching of arachnoid trabeculae, caused by the puncture needle. Their results might have important implications for epilepsy surgeons when planning SEEG [29].

Nakae S et al. also published a case report about the visualization of the vagal nerve using CT angiography and MRI for the implantation of a vagal nerve stimulator [30,31]. As they described, understanding the running course of the vagus nerve is very important for safe surgery. Usually, the vagus nerve runs between the common carotid artery and the internal jugular vein; however, in some cases, it runs below or above these vessels [32], and it takes surgeons much longer to detect the vagus nerve. They fused CT angiography and MRI obtained preoperatively and colored the vagus nerve. Their idea might lead to reductions in complication rates and surgical times for the implantation of vagus nerve stimulators [33].

Yamazaki K et al. published a case report about rare frontal encephaloceles presenting with epilepsy treated surgically. “Encephaloceles” are known to be associated with epilepsy, but mostly, they are located in the temporal lobe [34,35,36,37,38,39] and are rarely located in the frontal lobe [40,41]. The authors demonstrated the clear findings of the MRI, PET and electroencephalography of rare encephaloceles in frontal lobes and their successful surgical treatment via frontal lobectomy. Also, they reviewed previous research regarding encephaloceles with epilepsy. This report might be an important reference for surgeons when experiencing the same clinical situations [42].

I believe that all of the articles in this Special Issue are valuable and will have important implications for clinicians in relation to brain tumor surgery and epilepsy.

## References

[1] Candy N.G., Ovenden C., Jukes A.K., Wormald P.J., Psaltis A.J. (2023). The learning curve for endoscopic endonasal pituitary surgery: A systematic review. Neurosurg. Rev..

[2] Guinto G., Guinto-Nishimura G.Y., Sangrador-Deitos M.V., Uribe-Pacheco R., Soto-Martinez R., Gallardo D., Guinto P., Vargas A., Aréchiga N. (2023). Current and Future Perspectives of Microscopic and Endoscopic Transsphenoidal Surgery for Pituitary Adenomas: A Narrative Review. Arch. Med. Res..

[3] Borg A., Kirkman M.A., Choi D. (2016). Endoscopic Endonasal Anterior Skull Base Surgery: A Systematic Review of Complications During the Past 65 Years. World Neurosurg..

[4] Prevedello D.M., Ditzel Filho L.F., Solari D., Carrau R.L., Kassam A.B. (2010). Expanded endonasal approaches to middle cranial fossa and posterior fossa tumors. Neurosurg. Clin. N. Am..

[5] Ryvlin P., Rheims S., Hirsch L.J., Sokolov A., Jehi L. (2021). Neuromodulation in epilepsy: State-of-the-art approved therapies. Lancet Neurol..

[6] Uda T., Kunihiro N., Nakajo K., Kuki I., Fukuoka M., Ohata K. (2018). Seizure freedom from temporal lobe epilepsy with mesial temporal lobe tumor by tumor removal alone without hippocampectomy despite remaining abnormal discharges on intraoperative electrocorticography: Report of two pediatric cases and reconsideration of the surgical strategy. Surg. Neurol. Int..

[7] Yu H.Y., Lin C.F., Chou C.C., Lu Y.J., Hsu S.P.C., Lee C.C., Chen C. (2021). Outcomes of hippocampus-sparing lesionectomy for temporal lobe epilepsy and the significance of intraoperative hippocampography. Clin. Neurophysiol..

[8] Morioka T., Hashiguchi K., Nagata S., Miyagi Y., Yoshida F., Shono T., Mihara F., Koga H., Sasaki T. (2007). Additional hippocampectomy in the surgical management of intractable temporal lobe epilepsy associated with glioneuronal tumor. Neurol. Res..

[9] Gleissner U., Helmstaedter C., Schramm J., Elger C.E. (2002). Memory outcome after selective amygdalohippocampectomy: A study in 140 patients with temporal lobe epilepsy. Epilepsia.

[10] Morris H.H., Matkovic Z., Estes M.L., Prayson R.A., Comair Y.G., Turnbull J., Najm I., Kotagal P., Wyllie E. (1998). Ganglioglioma and intractable epilepsy: Clinical and neurophysiologic features and predictors of outcome after surgery. Epilepsia.

[11] Takayama Y., Ikegaya N., Iijima K., Kimura Y., Kosugi K., Yokosako S., Kaneko Y., Yamamoto T., Iwasaki M. (2022). Is Hippocampal Resection Necessary for Low-Grade Epilepsy-Associated Tumors in the Temporal Lobe?. Brain Sci..

[12] Ichinose T., Goto T., Ishibashi K., Takami T., Ohata K. (2010). The role of radical microsurgical resection in multimodal treatment for skull base meningioma. J. Neurosurg..

[13] Morisako H., Goto T., Ohata K. (2015). Petroclival meningiomas resected via a combined transpetrosal approach: Surgical outcomes in 60 cases and a new scoring system for clinical evaluation. J. Neurosurg..

[14] Goto T., Ohata K. (2016). Surgical Resectability of Skull Base Meningiomas. Neurol. Med. Chir..

[15] Schwartz T.H., Morgenstern P.F., Anand V.K. (2019). Lessons learned in the evolution of endoscopic skull base surgery. J. Neurosurg..

[16] Carnevale J.A., Pandey A., Ramirez-Loera C., Goldberg J.L., Bander E.D., Henderson F., Niogi S.N., Tabaee A., Kacker A., Anand V.K. (2023). Endonasal, supraorbital, and transorbital approaches: Minimal access endoscope-assisted surgical approaches for meningiomas in the anterior and middle cranial fossae. J. Neurosurg..

[17] Bove I., Cheok S., Ruzevick J.J., Zada G. (2023). Endoscopic Endonasal and Keyhole Surgery for Skull Base Meningiomas. Neurosurg. Clin. N. Am..

[18] Uda H., Uda T., Kinoshita M., Kishima H., Tanoue Y., Nagahama A., Kawashima T., Ohata H., Nakajo K., Morisako H. (2022). Visualization of Resected Area in Endonasal Endoscopic Approach versus Transcranial Approach for Skull Base Meningiomas by Voxel-Based-Lesion Mapping. Brain Sci..

[19] Ohue S., Kohno S., Inoue A., Yamashita D., Matsumoto S., Suehiro S., Kumon Y., Kikuchi K., Ohnishi T. (2015). Surgical results of tumor resection using tractography-integrated navigation-guided fence-post catheter techniques and motor-evoked potentials for preservation of motor function in patients with glioblastomas near the pyramidal tracts. Neurosurg. Rev..

[20] Fujii Y., Ogiwara T., Goto T., Kanaya K., Hara Y., Hanaoka Y., Hardian R.F., Hongo K., Horiuchi T. (2021). Microscopic Navigation-Guided Fence Post Technique for Maximal Tumor Resection During Glioma Surgery. World Neurosurg..

[21] Kajiwara K., Yoshikawa K., Ideguchi M., Nomura S., Fujisawa H., Akimura T., Kato S., Fujii M., Suzuki M. (2010). Navigation-guided fence-post tube technique for resection of a brain tumor: Technical note. Minim. Invasive Neurosurg..

[22] Yoshikawa K., Kajiwara K., Morioka J., Fujii M., Tanaka N., Fujisawa H., Kato S., Nomura And S., Suzuki M. (2006). Improvement of functional outcome after radical surgery in glioblastoma patients: The efficacy of a navigation-guided fence-post procedure and neurophysiological monitoring. J. Neurooncol..

[23] Nakae S., Kumon M., Teranishi T., Ohba S., Hirose Y. (2023). Applied Fence-Post Techniques Using Deep Electrodes Instead of Catheters for Resection of Glioma Complicated with Frequent Epileptic Seizures: A Case Report. Brain Sci..

[24] Mullin J.P., Shriver M., Alomar S., Najm I., Bulacio J., Chauvel P., Gonzalez-Martinez J. (2016). Is SEEG safe? A systematic review and meta-analysis of stereo-electroencephalography-related complications. Epilepsia.

[25] McGovern R.A., Ruggieri P., Bulacio J., Najm I., Bingaman W.E., Gonzalez-Martinez J.A. (2019). Risk analysis of hemorrhage in stereo-electroencephalography procedures. Epilepsia.

[26] Agashe S., Brinkmann B.H., Cox B.C., Wong-Kisiel L., Van Gompel J.J., Marsh R.W., Miller K.J., Krecke K.N., Britton J.W. (2023). Implications of intracranial hemorrhage associated with stereo-EEG. Clin. Neurophysiol..

[27] Willems L.M., Reif P.S., Spyrantis A., Cattani A., Freiman T.M., Seifert V., Wagner M., You S.J., Schubert-Bast S., Bauer S. (2019). Invasive EEG-electrodes in presurgical evaluation of epilepsies: Systematic analysis of implantation-, video-EEG-monitoring- and explantation-related complications, and review of literature. Epilepsy Behav..

[28] Miller C., Schatmeyer B., Landazuri P., Uysal U., Nazzaro J., Kinsman M.J., Camarata P.J., Ulloa C.M., Hammond N., Pearson C. (2021). sEEG for expansion of a surgical epilepsy program: Safety and efficacy in 152 consecutive cases. Epilepsia Open.

[29] Kawashima T., Uda T., Koh S., Yindeedej V., Ishino N., Ichinose T., Arima H., Sakuma S., Goto T. (2023). Intraparenchymal and Subarachnoid Hemorrhage in Stereotactic Electroencephalography Caused by Indirect Adjacent Arterial Injury: Illustrative Case. Brain Sci..

[30] Kawai K., Tanaka T., Baba H., Bunker M., Ikeda A., Inoue Y., Kameyama S., Kaneko S., Kato A., Nozawa T. (2017). Outcome of vagus nerve stimulation for drug-resistant epilepsy: The first three years of a prospective Japanese registry. Epileptic Disord..

[31] Penry J.K., Dean J.C. (1990). Prevention of intractable partial seizures by intermittent vagal stimulation in humans: Preliminary results. Epilepsia.

[32] Park J.K., Jeong S.Y., Lee J.H., Lim G.C., Chang J.W. (2011). Variations in the course of the cervical vagus nerve on thyroid ultrasonography. AJNR Am. J. Neuroradiol..

[33] Nakae S., Kumon M., Katagata A., Murayama K., Hirose Y. (2023). Vagus Nerve Visualization Using Fused Images of 3D-CT Angiography and MRI as Preoperative Evaluation for Vagus Nerve Stimulation. Brain Sci..

[34] Ramos-Fresnedo A., Domingo R.A., McGeary R.C., Sirven J.I., Feyissa A.M., Tatum W., Ritaccio A.L., Middlebrooks E.H., Grewal S.S. (2021). Encephalocele-Associated Drug-Resistant Epilepsy of Adult Onset: Diagnosis, Management, and Outcomes. World Neurosurg..

[35] Smith K.M., Kanth K.M., Krecke K.N., Alden E.C., Patel J.S., Witte R.J., Van Gompel J.J., So E., Britton J.W., Cascino G.D. (2023). Drug-resistant temporal lobe epilepsy with temporal encephaloceles: How far to resect. Epilepsy Behav..

[36] Agashe S., Lundstrom B.N., Brinkmann B.H., So E., Cascino G.D., Gregg N., Marsh W.R., Cross M., Van Gompel J.J., Smith K.M. (2023). Temporal encephalocele: An epileptogenic focus confirmed by direct intracranial electroencephalography. Epilepsy Behav. Rep..

[37] Buraniqi E., Guerin J.B., Miller K.J., Van Gompel J.J., Krecke K., Wirrell E.C., Nickels K.C., Payne E.T., Wong-Kisiel L. (2023). Temporal Encephalocele: A Treatable Etiology of Drug-Resistant Pediatric Temporal Lobe Epilepsy. Pediatr. Neurol..

[38] Samudra N., Armour E., Gonzalez H., Mattingly D., Haas K., Singh P., Sonmezturk H., Gallagher M., Crudele A., Nobis W. (2023). Epilepsy with anterior temporal encephaloceles: Baseline characteristics, post-surgical outcomes, and comparison to mesial temporal sclerosis. Epilepsy Behav..

[39] Jagtap S.A., Kurwale N., Patil S., Bapat D., Joshi A., Chitnis S., Deshmukh Y., Nilegaonkar S. (2022). Temporal encephalocele: A rare but treatable cause of temporal lobe epilepsy. Epileptic Disord..

[40] Atli B., Rath S., Burtscher J., Hainfellner J.A., Hametner S. (2022). Frontal intradiploic encephalocele in a 44-year-old male patient: Illustrative case. J. Neurosurg. Case Lessons.

[41] Faulkner H.J., Sandeman D.R., Love S., Likeman M.J., Nunez D.A., Lhatoo S.D. (2010). Epilepsy surgery for refractory epilepsy due to encephalocele: A case report and review of the literature. Epileptic Disord..

[42] Yamazaki K., Kanaya K., Uda T., Fukuyama T., Nishioka M., Hoshino Y., Kaneko T., Hardian R.F., Yamazaki D., Kuwabara H. (2023). Frontal Encephalocele Plus Epilepsy: A Case Report and Review of the Literature. Brain Sci..

